# Spatiotemporal Analysis of Overall Health in the United States Between 2010 and 2018

**DOI:** 10.7759/cureus.18295

**Published:** 2021-09-26

**Authors:** Binod Acharya, Loni Tabb

**Affiliations:** 1 Urban Health Collaborative, Drexel University, Philadelphia, USA; 2 Department of Epidemiology and Biostatistics, Drexel University, Philadelphia, USA

**Keywords:** bayesian statistics, county health rankings, health disparity, spatiotemporal, overall health

## Abstract

Background

Although many previous studies have documented spatial heterogeneity in health outcomes across the United States at different geographic scales, spatiotemporal analyses to understand overall health are scant.

Methodology

We used the County Health Rankings (CHR) data to analyze the three types of health outcomes, viz., overall health, length of life, and quality of life for 2010-2018 in the contiguous United States employing hierarchal Bayesian methods. Composite scores were created to proxy these outcomes utilizing predefined weights of several variables as recommended by CHR. Our methods assumed a convolution of spatially structured and unstructured errors to model the overall spatial error. Spatial effects were modeled using conditional autoregressive distribution.

Results

The substantial disparity in these health outcomes was evident, with counties having poorer health outcomes mostly concentrated in the southeastern United States. Models that incorporated county-level demographic and socioeconomic characteristics partially explained the observed spatial heterogeneity in health outcomes. Interestingly, there was no time effect in any of the outcomes suggesting a perpetuation of health disparity over the years.

Conclusions

County-specific health policy interventions that take into account the contextual factors might be beneficial in improving population health and breaking the perpetuation of health disparity.

## Introduction

A wide body of literature has documented the presence of spatial heterogeneity in health outcomes across the United States [1-4]. Such spatial heterogeneity in health outcomes has been examined with respect to disease-specific outcomes [5,6] or the overall population health [1] and quality of life [7].

Examples of well-documented spatial health disparity include poorer child health outcomes in the southern states compared to the northern states [2], a lower heart disease mortality rate in the northeast than in the south [8], and a higher concentration of diabetes prevalence in the southeastern counties [6]. Although the etiology behind the relationship between health outcomes and geography can be complex, the geographic disparity in health outcomes is often associated with the spatially patterned distribution of determinants of health such as differences in socioeconomic factors [9], health behaviors such as smoking, access to and quality of clinical care [10], and physical environment [11].

Most available studies in health disparity are cross-sectional in nature. These studies compare health outcomes among places and/or sociodemographic groups and provide a snapshot of health status at a fixed point in time. However, understanding the temporal trend of health outcomes is as important as understanding the spatial patterns of health outcomes. Different geographic regions may experience differential improvements (or deteriorations) in health outcomes over time. This calls for a joint space-time approach to better characterize the state of health.

The County Health Rankings (CHR) is a mutual endeavor of the University of Wisconsin Population Health Institute and the Robert Wood Johnson Foundation that aims to mobilize action toward community health by stimulating interest among the public and policymakers [12]. CHR has been producing state-level annual rankings for over 3,000 counties and county-equivalents since 2010. The availability of CHR data provides a unique opportunity to track the overall health across US counties over space and time. The standard CHR methodology provides a ranking of counties within each state for overall health outcomes and for four categories of health factors. The overall health outcome is a joint measure of the length of life (mortality) and quality of life. More specifically, the years of potential life lost before the age of 75 per 100,000 population proxies the measure of the length of life, while four additional sets of measures (self-reported fair or poor health prevalence, mean number of poor mental health days per month, mean number of poor physical health days per month, and percentage of live births with low birth weight) define the quality of life [12].

An extensive body of literature exists in the space-time modeling of health outcomes, particularly in the Bayesian disease mapping context. A popular approach to use in the Bayesian context is to employ the hierarchical models utilizing Markov Chain Monte Carlo (MCMC) methods [8,13-16]. A widely used approach for modeling areal data in this framework is conditional autoregressive (CAR) models. Several variants of the CAR distributions exist, including the simplest intrinsic CAR (ICAR) model and the convolution model [17,18]. The ICAR model only detects strong spatial correlation, whereas the convolution model provides additional flexibility to capture both spatially structured and unstructured random effects by incorporating two sets of random effects [19].

In this paper, we address the following questions: (a) how much spatiotemporal variability exists in the mortality, quality of life, and overall health outcome across the United States between 2010 and 2018?; and (b) can the spatial heterogeneity in health outcomes be explained by county-level demographic and socioeconomic characteristics, and to what extent?

## Materials and methods

Data sources

Yearly health outcomes data from 2010 to 2018 for 3,108 counties or county-equivalents in the contiguous United States were obtained from CHR and the roadmaps website (http://www.countyhealthrankings.org). Health outcomes data obtained included a single measure of the length of life and four measures of quality of life. Length of life is assessed by the years of potential life lost per 100,000 population before the age of 75 which CHR collects from the National Center for Health Statistics (NCHS)-mortality files. The following variables measure the quality of life: (a) the percentage of adults who report fair or poor health, (b) the average number of reported physically unhealthy days per month, (c) the average number of reported mentally unhealthy days per month, and (d) the percentage of births with low birth weight (<2,500 g). The CHR compiles data on poor or fair health, poor physical health days, and poor mental health days using the Behavioral Risk Factor Surveillance System (BRFSS), which is a cross-sectional telephone survey conducted by the Centers for Disease Control and Prevention (CDC) each year and the data on birthweight using NCHS-natality files.

The county-level socioeconomic and demographic variables were obtained from the American Community Survey (ACS) five-year estimates dataset for 2012-2016 housed at the Urban Health Collaborative, Drexel University. The ACS estimates roughly correspond to the mid-time of our study period, 2010-2018. After the evaluation of variance inflation factors, the following county-level demographic and socioeconomic covariates were included in the models: (a) percentage of the African American population, (b) percentage of the Hispanic population, (c) percentage of females, (d) percentage of the population aged ≥65 years, (e) percentage of the population aged <18, (f) percentage of the population with a minimum of bachelor-level education, (g) median household income, (h) population density, (i) poverty rate, (j) unemployment rate, and (k) GINI index of income inequality. These variables were standardized in our analysis.

Development of composite z-score

We calculated standardized scores for each of the five measures (one measure for the length of life and four measures for the quality of life) relative to all 3,108 counties that were being studied. We call that standardized score a z-score such that: $z=\frac{County\ value-Average\ of\ Counties}{Standard\ deviation\ of\ Counties}.$

Before calculating z-scores, we imputed the state-level means for each of the five individual measures corresponding to the length and quality of life for a given year when the data were missing, following the standard CHR protocol that imputes state-level means to rank counties within a state [13]. The most frequent missing values were for mentally unhealthy days per month, where 17.66% of counties lacked this information for 2012-2015.

Following the CHR protocol, z-scores that were <−3.0 and >3.0 in counties with a population of less than 20,000 were truncated to −3.0 and 3.0, respectively. This accounts for the possibility that very high or very low z-scores in counties with smaller populations could just be the statistical artifact of having smaller sample sizes (or smaller health events to report) rather than their true values. These z-scores are unit-free and relative to all counties under study, allowing us to combine them into summary indices.

Based on the z-scores for five variables described above, we calculated two additional composite z-scores: (a) composite z-score for quality of life and (b) composite z-score for overall health outcome. The composite z-score of quality of life was calculated with four individual scores (low birth weight score was given a weight of 40%, and poor or fair health, poor physical health days, and poor mental health days scores were each given a weight of 20%). The composite z-score for overall health outcome was calculated by combining five individual z-scores with weights predefined by CHR (the length of life score was given a weight of 50%, low birth weight score was given a weight of 20%, and poor or fair health, poor physical health days, and poor mental health days scores were each given a weight of 10%). The composite z-scores were standardized for each year, such that they had a mean of 0 and a standard deviation of 1. We base our further analysis on three types of z-scores: (a) composite z-score for overall health outcome, (b) composite z-score for quality of life, and (c) z-score for the length of life.

Statistical analysis

We model the z-scores for overall health, length of life, and quality of life separately via Gaussian likelihood.

(1) \\Y_{ij} \sim~ Normal\ (\mu_{ij},\sigma_j^2)\\,

where $Y_{ij}$ denotes the observed z-score for county $i=1,\dots,3108$ and year $j=2010,\dots,2018$. We fitted two sets of models: the first set without demographic and socioeconomic variables, which we refer to as null models, and the second set with demographic and socioeconomic factors, which we refer to as fully adjusted models.

We fitted three individual null models that model spatial variability in the same way but differ in ways to capture temporal variability. To capture the spatial effects, the county-specific spatial error term was decomposed into county-specific spatially unstructured zero-mean normally distributed random error $u_i$ and spatially structured random effects (clustering/correlated error) $b_i$.

Our null models take the following forms:

(2a) $\mu_{ij}=\alpha\ +u_i+b_i+\beta_t\times t_j$

(2b) $\mu_{ij}={\alpha\ +u}_i+b_i+\beta_t\times t_j+\phi_j$

(2c) $\mu_{ij}={\alpha\ +u}_i+b_i+\phi_j+\psi_j$

In model 2a, we introduce fixed time effect $\beta_t$. In model 2b, we introduce additional temporally unstructured random effects $\phi_j$ in addition to the fixed time effect. In model 2c, we introduce temporally structured $\psi_j$ and unstructured random effects $\phi_j$ to capture the overall temporal trend where the structured error is modeled via an autoregressive random walk function.

Our fully adjusted model includes county-level demographic and socioeconomic variables. In addition to the parameters in null models described above, our fully adjusted model included the fixed-effect terms for each of these socioeconomic and demographic covariates. In equations 3a-3c, $X_i$ denotes the vector of county-level demographic and socioeconomic covariates with the corresponding regression coefficient vector, β.

(3a) $\mu_{ij}=\alpha\ +u_i+b_i+\beta_t\times t_j + \mathbf X_i^T\beta$

(3b) $\mu_{ij}=\alpha\ +u_i+b_i+\beta_t\times t_j + \phi_j + \mathbf X_i^T\beta$

(3c) $\mu_{ij}=\alpha\ +u_i+b_i+\beta_t\times t_j + \phi_j + \psi_j + \mathbf X_i^T\beta$

We modeled the precision parameter from (1) with a weakly informative gamma (0.05, 0.05) prior. Parameters controlling the mean effect were modeled with the prior distributions, as described below. Priors for spatially structured random effects were assigned through an ICAR distribution. Under the ICAR distribution, the mean of a county depends on neighboring counties. A variance parameter dictates the variability of random effects, conditional upon the effects in neighboring areas. For simplicity, we define counties $i$ and $k$ as neighbors, if they share one or more common vertex between boundaries, commonly referred to as Queen’s contiguity. For each county, the expected value of $b_i$ is the mean of its neighboring counties, and the variance of $b_i$ is inversely proportional to the number of neighbors for that county, $m_i$

$b_i\ |\ b_{k,\ k\neq i\ }\sim\ Normal\ \left({\bar{b}}_i,\ \frac{\sigma_b^2}{m_i}\right)$,

where $\bar{b_i} = \sum_{k,k\neq i}\frac{w_{ik} b_k}{m_i}$ and $\\W=\{w_{ik}\}$ is $3108 \times 3108$ adjacency matrix with elements $w_{i,k}=1$ if counties $\\i$ and $\\k$ are neighbors and 0 otherwise. For spatially unstructured random effects $\\u_i$ we assume an exchangeable normal prior with a mean of zero and precision of $\tau_u$.

The fixed-effect coefficient of temporal effect, $\beta_t$ in models (2a, 3a, 2b, 3b) was assigned normal prior with mean 0 and the precision $\tau_t$. The unstructured temporal effect $\phi_j$ in models (2b, 3b) is modeled with a normal prior with mean 0 and precision $\tau_\phi$. The precision parameters $\tau_u , \tau_t, \tau_\phi$ were modelled with weakly informative gamma (0.05, 0.05) hyperpriors. We modeled structured temporal autocorrelation in models (2c, 3c) using an autoregressive random walk prior of order 1 where the mean parameter equals the mean effect from the preceding year and the precision parameter receives a noninformative gamma hyperprior. Noninformative normal priors were assigned to each of the regression coefficients corresponding to demographic and socioeconomic variables. The intercept α was assigned an improper uniform prior.

For each model, two parallel chains were run for 5,000 iterations with an update-level thinning of 100 using the software WinBUGS. Convergence was monitored within WinBUGS by visual examination of the trace plots of the sample for each chain, autocorrelation plots, and the Gelman-Rubin plots. The first 1,000 iterations were discarded as burn-in. Deviance Information Criterion (DIC), a Bayesian measure of model fit penalized for complexity, was used to identify the better fitting model [20,21].

## Results

Among the different models considered, the models with the fixed effect for the time (models 2a and 3a) had the lowest DIC for both null and adjusted types and for all three types of health outcomes (overall health, length of life, and quality of life), indicating a better fit. We base our inferences on these models in the rest of the paper.

Table 1 presents the change in the precision estimates of spatial error terms from null to fully adjusted models.

**Table 1:** Precision parameters of spatial effects from null and fully adjusted models.

Parameter	Model type	Median (95% credible interval)	Percentage increase in mean precision
Overall health			
Structured spatial effect	Null	1.12 (1.00, 1.27)	144.6
	Fully adjusted	2.74 (2.40, 3.14)	
Unstructured spatial effect	Null	9.20 (7.76, 10.98)	200.5
	Fully adjusted	27.65 (22.02, 36.16)	
Length of life			
Structured spatial effect	Null	0.94 (0.83, 1.08)	138.3
	Fully adjusted	2.24 (1.91, 2.63)	
Unstructured spatial effect	Null	7.32 (6.12, 8.89)	112.6
	Fully adjusted	15.56 (12.75, 19.78)	
Quality of life			
Structured spatial effect	Null	1.42 (1.25, 1.63)	85.9
	Fully adjusted	2.64 (2.28, 3.07)	
Unstructured spatial effect	Null	9.24 (7.91, 10.90)	72.5
	Fully adjusted	15.94 (13.46, 19.26)	

There is a substantial reduction in the variance of spatial error estimates in the full models, particularly for the overall health outcome due to the attenuation of spatial errors.

**Figure 1:**
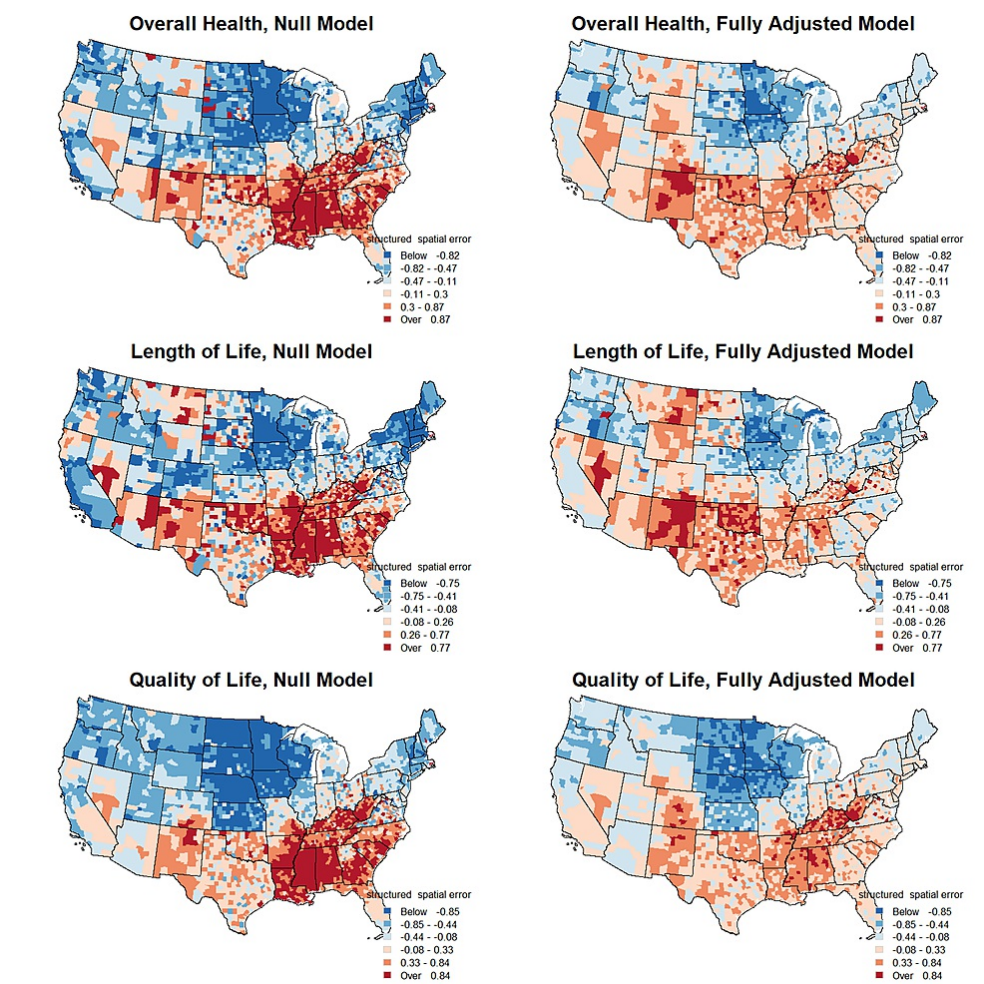
Spatially structured errors for overall health, length of life, and quality of life from null and fully adjusted models.

A significant spatial correlation in overall health, length of life, and quality of life is evident from our models which can be visualized by mapping the county-specific spatially structured error terms (Figure 1).

The clustering of spatial errors translates into the spatial pattern of composite z-scores suggesting clusters of counties with poorer health outcomes in the southeastern and southwestern United States, which contrasts with the counties in the midwest having relatively better outcomes. We observed palpably smaller spatially structured errors in fully adjusted models compared to null models for all three types of outcomes (Figure 1). This attenuation of spatial effects is pronounced in many counties in the southeast and midwest where there is a notable departure in spatial effects from null models to adjusted models.

The reduction of spatial errors in full models compared to null models is unsurprising as most of the socioeconomic and demographic variables included in the fully adjusted models were significant, as evident by 95% credible intervals of posterior estimates not including zero (Table 2).

**Table 2:** Estimates of fixed-effect parameters for overall health, length of life, and quality of life from fully adjusted models. CrI: credible interval

Covariates	Overall health, Median (95% CrI)	Length of life, Median (95% CrI)	Quality of life, Median (95% CrI)
% African Americans	0.168 (0.141, 0.197)	0.097 (0.064, 0.130)	0.208 (0.178, 0.240)
% Minimum bachelor’s education	-0.256 (-0.282, -0.230)	-0.256 (-0.286, -0.225)	-0.238 (-0.266, -0.209)
% Females	0.045 (0.029, 0.062)	0.038 (0.018, 0.058)	0.052 (0.033, 0.070)
GINI Index	0.035 (0.017, 0.054)	0.051 (0.029, 0.073)	0.015 (-0.005, 0.037)
% Hispanic	-0.132 (-0.162, -0.103)	-0.223 (-0.259, -0.189)	-0.010 (-0.043, 0.022)
Median household income	-0.143 (-0.179, -0.106)	-0.149 (-0.192, -0.105)	-0.093 (-0.134, -0.053)
% Population ≥65 years	0.044 (0.021, 0.068)	0.099 (0.072, 0.127)	-0.037 (-0.063, -0.010)
% Population <18 years	0.063 (0.039, 0.087)	0.125 (0.096, 0.146)	-0.040 (-0.066, -0.013)
Population density	0.030 (0.011, 0.050)	0.031 (0.008, 0.054)	0.026 (0.005, 0.048)
Poverty rate	0.180 (0.151, 0.212)	0.186 (0.152, 0.224)	0.125 (0.092, 0.162)
% Unemployment	0.097 (0.076, 0.117)	0.121 (0.096, 0.146)	0.054 (0.029, 0.076)
Time	~0 (-0.001, 0.001)	~0 (-0.002, 0.002)	~0 (-0.012, 0.012)

This suggests that these variables are important determinants of the state of health in the United States. Still, the smoothening in full models remains far from perfect; for example, there are counties in New Mexico that stand out where the smoothening appears to be inadequate. Moreover, there remained a substantial clustering of health outcomes in the southeast even after accounting for the socioeconomic and demographic characteristics of the counties.

Interestingly, we did not see a temporal trend in the length or quality of life, overall health, and across US counties, despite using longitudinal data for nine years, as indicated by the coefficients for time effect very close to zero. The persistence of a similar pattern across years suggests that there is no notable relative improvement or deterioration in health across US counties.

Table 2 presents the estimated regression coefficients for county-level socioeconomic and demographic factors from fully adjusted models for overall health, length of life, and quality of life composite scores. Among the covariates in our models, education was the most important predictor of overall health, length of life, and quality of life. For instance, one standard deviation increase in the proportion of people with a minimum of bachelor’s education, while controlling for other covariates, would result in about 0.26, 0.26, and 0.24 unit decrease in the composite z-scores for overall health, length of life, and quality of life, respectively, indicative of improved health outcomes. On the other hand, one standard deviation increase in the county-level poverty rate would increase the composite z-score for overall health, length of life, and quality of life by 0.18, 0.19, and 0.13 units, respectively, while accounting for other covariates, indicative of declining health outcomes.

Moreover, the proportion of African American population was negatively associated with health outcomes. Specifically, one standard deviation increase in the proportion of the African American population, while controlling for other covariates, would result in about 0.17, 0.10, and 0.21 unit increase in overall health, length of life, quality of life composite z-scores, respectively. Interestingly, we noticed that the percentage of the Hispanic population was significantly associated with improved length of life and overall health but no association with quality of life.

A higher proportion of females appears to be associated with poor health for all three types of outcomes. The proportion of the senior population aged 65 years or older and the proportion of the young population aged 18 years or younger appear to be negatively associated with overall health and length of life but they were positively associated with quality of life. In all three health outcomes we examined, the median household income of counties was positively associated with improved health. County-level unemployment rate and population density appeared to be negatively associated with all types of health outcomes.

## Discussion

From our results, we saw a notable spatial heterogeneity in overall health, length of life, and quality of life across the contiguous US counties. Many southern counties fared worse than their midwest counterparts even after adjusting for county-level demographic and socioeconomic characteristics. In general, the counties with poorer health correspond to the Coronary Valley [8], Stroke Belt [22], and Diabetes Belt [23]. In addition, we found that demographic and socioeconomic characteristics of counties are useful (and most of the time, significant) in understanding the spatial health disparity. Evidence of substantial clustering of health outcomes from adjusted models, although lower in magnitude than from unadjusted models, indicates that there could be unmeasured confounders beyond the socioeconomic factors we included in our models or the “intrinsic” effect of place or both.

Whereas we understand that the nature of health disparities can be complex and intersectional, we contend that our findings are broadly in line with past literature on health disparity in the United States. Many previous studies have established a positive association between income and higher education and better health outcomes in the individual, community, or larger social context [9,24]. Poverty and unemployment, on the other hand, have long been linked to poorer health outcomes in health disparity literature. Our findings corroborate these established findings. As we learn from the results, the racial composition of counties is an important indicator of county-level health outcomes suggesting the prevalence of racial disparities in terms of health outcomes. We found a positive association between the proportion of the Hispanic population and overall health but no association with quality of life, which seems to support the idea of the Hispanic paradox prevalent in health disparity literature [25].

Interestingly, this spatial trend persisted throughout the study period indicative of no relative improvement or deterioration in health outcomes over time. However, because of the nature of the study, we could only model the relative improvements in health outcomes, not the absolute improvement. Moreover, the overall health and quality of life outcomes are “composite” measures derived from several other individual variables. Thus, there remains a possibility of meaningful time effects in individual components but in the opposite direction, eventually canceling each other out rendering no net effect from the higher-level composite variables we examined.

Limitations

There are a few limitations in this study that are worth mentioning. One of the limitations of the study is that there remains a substantial lag between CHR data reported for a given year and the years when original data were collected. For example, length of life data for the year 2010 came from NCHS Vital statistics data, 2004-2006. This implies that we were unable to model the health outcomes in “real-time.” Furthermore, CHR data for the length of life for 2013 and 2014 came from the same NCHS mortality files, 2008-2010. We also note that the measures that come from BRFSS are subject to recall bias of respondents.

## Conclusions

In summary, in line with the “place matters for health” narrative in health disparity-based literature, our findings demonstrate a presence of spatial disparity in health outcomes that can be partially explained by county-level demographic and socioeconomic characteristics. We also found persistence of spatial patterns in health outcomes over time. Although there have been efforts to improve health, the perpetuating regional health disparity shows a rather worrying picture. It perhaps indicates that area-specific interventions for equitable and just health are needed. County-specific interventions that take into account, not just the individual characteristics but also contextual factors might be beneficial in improving population health. Future research should focus on assessing the spatiotemporal trend of individual variables that were used to generate our composite scores to verify if they exhibit the same trend as composite scores.
