# Correction: Spatiotemporal Analysis of Overall Health in the United States Between 2010 and 2018

**DOI:** 10.7759/cureus.c56

**Published:** 2021-12-17

**Authors:** Binod Acharya, Loni Tabb

**Affiliations:** 1 Urban Health Collaborative, Drexel University, Philadelphia, USA; 2 Department of Epidemiology and Biostatistics, Drexel University, Philadelphia, USA

This article has been corrected to include Dr. Loni Tabb as second author. Dr. Tabb was erroneously omitted from the article due to a misunderstanding by the first author, Binod Acharya, regarding what constitutes authorship. Both Mr. Acharya and Dr. Tabb have agreed to this formal correction adding Dr. Tabb as second author. Additionally, the acknowledgement of Dr. Tabb has now been removed. 

